# Novel synthetic nucleotides of notifiable dengue (1–4), Japanese encephalitis, yellow fever and Zika flaviviruses

**DOI:** 10.4155/fsoa-2018-0081

**Published:** 2018-11-13

**Authors:** Gerry Amor Camer, Yuki Oikawa, Hitomi Omaki, Daiji Endoh

**Affiliations:** 1 University of Eastern Philippines, Department of Clinical Sciences - College of Veterinary Medicine, Catarman, Northern Samar 6400 Philippines; 2 Rakuno Gakuen University, Department of Radiation Biology-School of Veterinary Medicine, Ebetsu, 069-8501 Hokkaido, Japan

**Keywords:** algorithm, flaviviruses, oligomer, overlap-extension PCR, synthetic nucleotides

## Abstract

**Aim:**

To produce synthetic nucleotides of notifiable dengue virus (1–4 types), Japanese encephalitis, yellow fever and Zika flaviviruses. These notifiable flaviviruses, particularly dengue and Zika, are problematic mosquito-borne infections in the Philippines, as well as in those countries with tropical and subtropical climates.

**Method:**

An algorithmic design formulation of overlap extension – polymerase chain reaction (OE-PCR) was performed to propagate 50–60 oligomer lengths of select notifiable flaviviral RNAs to DNA nucleotides via the two-step process of OE-PCR.

**Result:**

Algorithmic OE-PCR design formulation efficiently produced 253–256 bp of notifiable flaviviruses. Comparing the newly designed algorithmic OE-PCR with existing executable programs demonstrated it to be efficient and useful in generating accurate sequences of synthetic flaviviral nucleotides.

**Conclusion:**

The efficiently and accurately produced novel synthetic nucleotides of notifiable dengue virus 1–4, Japanese encephalitis, yellow fever and Zika flaviviruses using OE-PCR is useful in understanding the dynamics of flaviviral species and holds potential for the development of synthetic nucleotide-based immunogens.

## Lay abstract

Dengue virus (1–4), Japanese encephalitis and Zika fevers are notable mosquito-borne infections that continue to be problematic in the Philippines and its southeast Asian neighbors. Flaviviral yellow fever affects other tropical countries. This study aimed to effectively and efficiently synthesize flaviviral nucleotides through a predictively accurate algorithmic overlap extension-polymerase chain reaction design. The cost-saving arithmetic formulation has enabled the advanced production of 253–256 bp flaviviral nucleotide lengths. The newly synthesized and sequenced products are now ready for further experimentation and open the door for immunological exploration.

## Graphical abstract

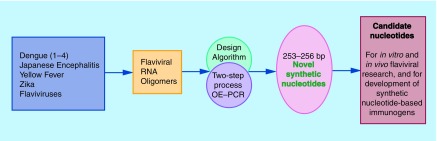

Dengue and Zika fevers, including Japanese encephalitis (JEV), are particularly burdensome mosquito-borne infections in the Philippines and are also selectively reported in Japan [1–4]. The challenges these diseases pose and the need to efficiently convert these notable flaviviruses into larger DNA fragments are evident, considering that the performance of currently developed vaccines and DNA-based immunogens remain imperfect [5,6]. Molecular-based methodologies are presently available in the literature with varying degrees of precision and accuracy for PCR amplification, disease detection, genetic probing and DNA construction [7,8]. Many artificial genes are now commercially produced by several manufacturers. Moreover, the inherent risks, including the biosecurity concerns associated with handling and producing DNA fragments of pathogenic viruses, as well as some highly virulent bacteria, delimit its commercialized propagation [4,9,10]. Depending on the type of oligomer being used, the cost–effectiveness of oligomer-size fragments varies. One of the promising modes for developing larger molecules of DNA is through the use of the overlap-extension polymer chain reaction (OE-PCR) method, which produces DNA fragments larger than 100 bp in size using synthetic oligomers [9,11,12]. The concerns that may have contributed to a rise in the price of oligomers have encouraged the development of novel model templates for PCR primers that can serve as a new application tool for OE-PCR. However, the oligomer design for OE-PCR may be problematic. This is because of homogeneity of the annealing temperature of the overlapping regions as well as the sense–antisense design of sequences [11,12]. As for positive controls for PCR detection, many DNA fragments may be produced; therefore, an algorithmic or automated design of oligomers for OE-PCR is indispensable [12]. Using an algorithmic OE-PCR design, this study was performed to efficiently propagate synthetic nucleotides of select flaviviruses [3,4]. Nucleotides of notifiable flaviviruses, namely dengue (1–4), JEV, yellow fever (YF) and Zika fevers, were efficiently and cost-effectively synthesized without the use of a DNA template, hence, generating synthetic select flaviviral DNAs useful for further viral studies and immunological explorations.

## Materials & methods

### The select list of notifiable flaviviral pathogens

Table 1 & Box 1 show the profiles of the seven selected flaviviruses (Eurofins Genomics, Co. Limited, Ota-ku Tokyo, Japan) for amplification using OE-PCR.

**Table 1.** **Profile of the seven notifiable flaviviral species.**

**Coding number**	**Flavivirus species**	**Accession number**	**Start**	**End**
2	Dengue virus 2	NC_001474.2	9157	9388

3	Dengue virus 4	NC_002640.1	9150	9381

5	Dengue virus 1	NC_001477.1	9158	9389

8	Dengue virus 3	NC_001475.2	9149	9380

15	Zika virus	NC_012532.1	9245	9479

25	Yellow fever virus	NC_002031.1	9227	9461

27	Japanese encephalitis virus	NC_001437.1	9270	9504

The OE-PCR in this experiment was performed via two processes: the first one is using the reference or original OE-PCR protocol [11,12] and the next one is the modified version of the OE-PCR. This was done to ensure optimization of deriving the desired amplified products.

### Reference/original overlap extension-polymerase chain reaction

1. Preparation of the first-stage primer mixed solution
  (A) Both ends primer (1, 6) 2.5 μl
  (B) Central primer (2–5) 0.5 μl
  (U) Deionized water (DW) 18 μl
2. Preparation of reaction solution for the first-step PCR
  (A) First-stage primer mixed solution × 6 μl
  (A) GoTaq 12.5 μl
  (U) DW 6.5 μl
3. First-stage PCR reaction
  (A) 95°C 2 min
  21
  (C) 72°C - 2 min
4. Preparation of the second-step PCR solution
  (A) First-step PCR reaction solution 1 μl
  (B) Both ends primer (stock solution) 1 μl × 2
  (C) GoTaq 12.5 μl
  (D) 9.5 μl of DW
5. Second-step PCR reaction
  (A) 95°C 2 min
  (A) (95°C - 1 min → 72°C - 2 min) × 35-times (C) 72°C - 2 min

### Modified overlap extension-polymerase chain reaction (OE-PCR)

1. Preparation of the first-stage primer-mixed solution

(A) Both ends primer (1, 6) 2.5 μl

(B) Central primer (2–5) 0.5 μl

(U) DW 193 μl

(A) GoTaq 12.5 μl

(U) DW 6.5 μl

3. First-stage PCR reaction

(A) 95°C 2 min

(A) (95°C - 1 min → 48°C - 1 min → 72°C - 1 min) × 20

(C) 72°C - 2 min

4. Preparation of the second-step PCR solution

(A) First-step PCR reaction solution 1 μl

(B) Both ends primer (stock solution) 1 μl × 2

(C) GoTaq 12.5 μl

(D) 9.5 μl of DW

5. Second-step PCR reaction

(A) 95°C 2 min

(B) (95°C. -1 min → 72°C. -2 min) × 25

(C) 72°C. -2 min

**Figure 1.**
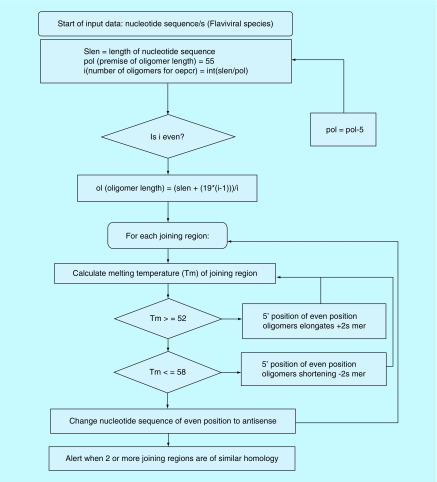
**Overlap extension-polymerase chain reaction design algorithm.** This formulation was prepared to algorithmically plan and accurately calculate the desired product for OE-PCR. The process was initially done using manual algorithm whereby executable OE-PCR oligomers can be designed on the limitation of user-determined oligomer length. The platform made use of Linux (Ubuntu). OE-PCR: Overlap extension-polymerase chain reaction.

Depicted in Figure 1 is the principle and process of OE-PCR as applied in this experiment.

In the second PCR, the most outside oligomers work for PCR primers and amplified the whole size of target DNA.

**Figure 2.**
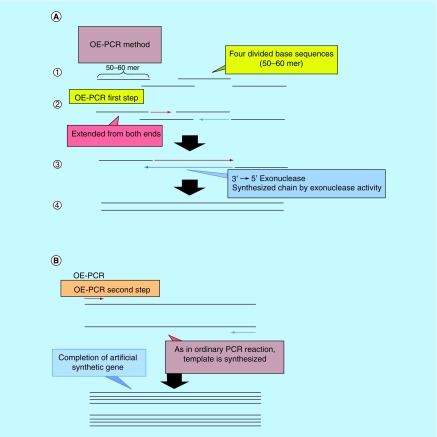
**Diagram of the two-step cycle overlap extension-polymerase chain reaction (A: first step; B: second step).** 1st PCR: For OE-PCR, 50–60mer oligomers were designed to have 19–23mer overlapping regions and make partial dimers **(a).** At the start of extension step of 1^st^ PCR, DNA was synthesized by polymerase which has a 3′→5′ exonuclease activity **(b).** From the exonuclease activity, some oligomers were digested and replaced by synthesized DNA strand **(c).** Whole length of DNA was synthesized **(d)**. OE-PCR: Overlap extension-polymerase chain reactions.

An electrophoresis of OE-PCR products was done for PCR product-size prediction after amplification of both OE-PCR and pathogen-specific PCR. Single product was confirmed on 1.5% agarose gel electrophoresis with 1 × Tris/Borate/EDTA (TBE) buffer and UV-visualized with 0.7 micro-g/ml-ethidium bromide (Figure 2). The PCR product sizes were determined by comparison with DNA molecular weight marker whereby sizes of the products estimated are referred to the 100 bp-ladder (Dooh Chemical Co., Ltd, Sapporo, Japan).

## Results & discussion

### Stepwise performance of overlap extension-polymerase chain reaction

**Figure 3.**
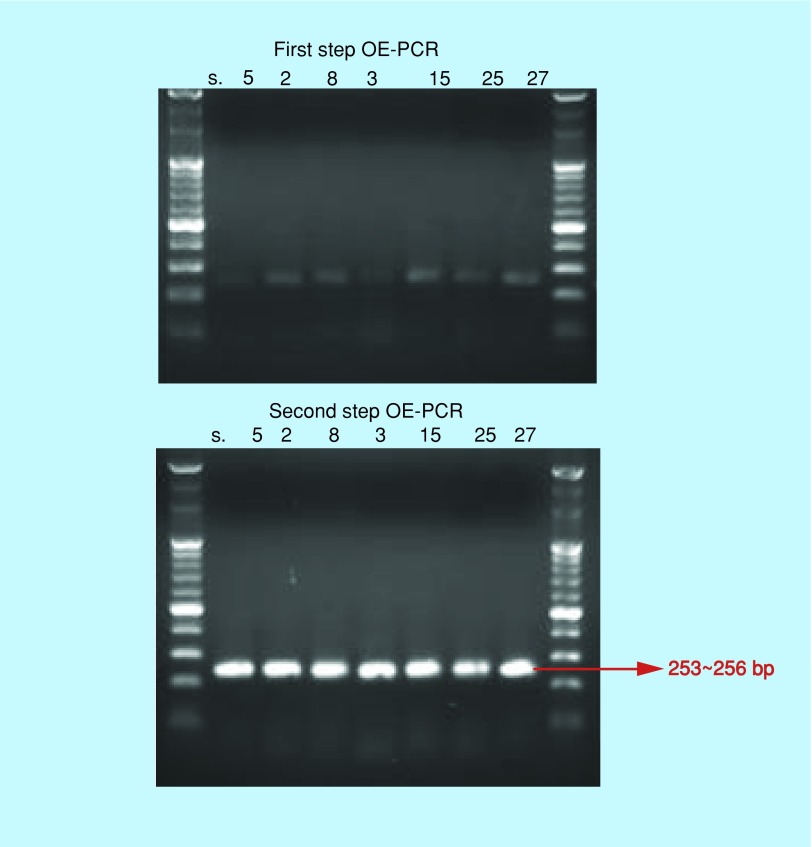
**First and second step overlap extension-polymerase chain reaction showing prominent DNA bands.** Note the almost similar product sizes of 253–256 bp. Each of the four Dengue virus has 253 bp; Zika, yellow fever and Japanese encephalitis has 256 bp DNAs. OE-PCR: Overlap extension-polymerase chain reactions.

The following notable flaviviruses, namely dengue (4 types), YF, JEV and Zika, were specifically processed for production of artificial DNAs. The algorithmic formulated OE-PCR was subsequently carried out in two-step cycles, with each cycle's DNA products depicted via UV-illuminated bands following gel electrophoresis (Figure 3). In the preliminary trials using this algorithmic OE-PCR, it was observed that a high success rate was derived when the number of oligomers was six or less compared with using an 8-oligomer-set or more. Thus, when OE-PCR product was used as a positive control, the ability to design PCR primers that produce about 100 bases in the product is required. This is possible at around 250 bp long and with algorithmic precision a well-synthesized 253–256 bp of DNA bands was yielded.

DNA sequencing was carried out in similar steps as previously described [1,7] (Eurofins Genomics, Co. Ltd, Ota-ku Tokyo). The newly sequenced products were subsequently registered at DDBJ/NCBI Gene Bank (see Table 2).

**Table 2.** **Genomic profile of newly synthesized dengue virus 1–4, Japanese encephalitis, yellow fever and Zika flaviviruses.**

**Number**	**DDBJ/NCBI gene bank accession number**	**Flavivirus species**	**Complete genome**	**Nucleotide sequence (synthetic)**
1	LC227563	Dengue virus 1	9158–9410	ATGTATGCAGATGACACAGCCGGATGGGACACAAGAATAACAGAGGATGATCTTCAGAATGAGGCCAAAATCACTGACATCATGGAACCTGAACATGCCCTATTGGCCACGTCAATCTTTAAGCTAACCTACCAAAACAAGGTAGTAAGGGTGCAGAGACCAGCGAAAAATGGAACCGTGATGGATGTCATATCCAGACGTGACCAGAGAGGAAGTGGACAGGTTGGAACCTATGGCTTAAACACCTTCACCA

2	LC227561	Dengue virus 2	9157–9409	ATGTATGCCGATGACACCGCAGGATGGGATACAAGAATCACACTAGAAGACCTAAAAAATGAAGAAATGGTAACAAACCACATGGAAGGAGAACACAAGAAACTAGCCGAGGCCATTTTCAAACTAACGTACCAAAACAAGGTGGTGCGTGTGCAAAGACCAACACCAAGAGGCACAGTAATGGACATCATATCGAGAAGAGACCAAAGAGGTAGTGGACAAGTTGGCACCTATGGACTCAATACTTTCACCA

3	LC227562	Dengue virus 3	9149–9401	ATGTATGCTGATGACACAGCTGGTTGGGACACAAGAATAACAGAAGATGACCTGCACAATGAGGAAAAGATCATACAGCAAATGGACCCTGAACACAGGCAGTTAGCGAACGCTATATTCAAGCTCACATACCAAAACAAAGTGGTCAAAGTTCAACGACCGACTCCAACGGGCACGGTAATGGATATTATATCTAGGAAAGACCAAAGGGGCAGTGGACAACTGGGAACTTATGGCCTGAATACATTCACCA

4	LC227568	Dengue virus 4	9150–9402	ATGTATGCTGATGACACAGCAGGCTGGGACACAAGAATCACTGAGGATGACCTTCAAAATGAGGAACTGATCACGGAACAGATGGCTCCCCACCACAAGATCCTAGCCAAAGCCATTTTCAAACTAACCTATCAAAACAAAGTGGTGAAAGTCCTCAGACCCACACCGCGGGGAGCGGTGATGGATATCATATCCAGGAAAGACCAAAGAGGTAGTGGACAAGTTGGAACATATGGTTTGAACACATTCACCA

5	LC227560	Japanese encephalitis virus	9270–9525	ATGTACGCTGATGACACCGCCGGATGGGACACTAGAATTACCAGAACTGATTTAGAAAATGAAGCCAAGGTGCTGGAGCTCCTAGACGGTGAACACCGCATGCTCGCTCGAGCCATAATTGAACTGACTTACAGGCACAAAGTGGTCAAGGTCATGAGACCTGCAGCAGAAGGAAAGACCGTAATGGACGTGATATCAAGAGAAGATCAAAGGGGGAGTGGACAGGTGGTCACTTATGCCCTTAACACTTTCACGA

6	LC227567	Yellow fever virus	9227–9482	TTCTACGCGGATGACACCGCTGGATGGGACACGCGCATCACAGAGGCAGACCTTGATGATGAACAGGAGATCTTGAACTACATGAGCCCACATCACAAAAAACTGGCACAAGCAGTGATGGAAATGACATACAAGAACAAAGTGGTGAAAGTGTTGAGACCAGCCCCAGGAGGGAAAGCCTACATGGATGTCATAAGTCGACGAGACCAGAGAGGATCCGGGCAGGTAGTGACTTATGCTCTGAACACCATCACCA

7	LC227589	Zika virus	9245–9500	ATGTACGCAGATGACACTGCTGGCTGGGACACCCGCATTAGTAAGTTTGATCTGGAGAATGAAGCTCTGATTACCAACCAAATGGAGGAAGGGCACAGAACTCTGGCGTTGGCCGTGATTAAATACACATACCAAAACAAAGTGGTGAAGGTTCTCAGACCAGCTGAAGGAGGAAAAACAGTTATGGACATCATTTCAAGACAAGACCAGAGAGGGAGTGGACAAGTTGTCACTTATGCTCTCAACACATTCACCA

DDBJ: DNA Data Bank of Japan; NCBI: National Center for Biotechnology Information.

Approaches to determine efficiency and cost–effectiveness of the current algorithmic OE-PCR tool versus the existing programs are presented in Table 3. These data delineate the utilized platform, the license, the program execution, the problems encountered in oligomer design and the methods of assembly [13–18].

**Table 3.** **Comparative features of oligomer design and assembly methods for overlap extension-polymerase chain reaction.**

**Program name**	**Study (year)**	**Platform**	**License**	**Execute**	**Problem**	**Ref.**
Speedy genes	Currin A, Swainston N, Day P, & Kell D. SpeedyGenes: exploiting an improved gene synthesis method for the efficient production of synthetic protein libraries for directed evolution. In Synthetic DNA Humana Press, New York, NY, 63–78 (2017)	Web (http://g.gene-genie.appspot.com/?)	Free	OK	Design limitation from AA sequence	[13]

	Kalendar R, Lee D and Schulman A. Java web tools for PCR, in silico PCR and oligonucleotide assembly and analysis. Genomics 98(2), 137–144 (2011) Kalendar R, Tselykh T, Khassenov B, & Ramanculov E. Introduction on using the FastPCR software and the related Java web tools for PCR and ligonucleotide assembly and analysis. PCR: Methods and Protocols, 33–64 (2017)	Windows/MacOS/Linux/Web (http://primerdigital.com/fastpcr.html)	Free (web)/charge	OE-PCR oligomer can be designed	Some oligomer exceeds its size	[14]

SITICHER2.0	O'Halloran D, Uriagereka-Herburger I and Bode K. STITCHER 2.0: primer design for overlapping PCR applications. Sci. Rep. 7:45349 (2017)	Web (http://www.ohalloranlab.net/STITCHER_2_0/index.html)	Free	Mainly for designing PCR-primers	No function for OE-PCR	[16]

Primer mapper	O'Halloran D. PrimerMapper: high throughput primer design and graphical assembly for PCR and SNP detection. Sci. Rep. 6:20631 (2016)	Linux (Perl)	Free	Mainly for designing PCR-primers	No function for OE-PCR	[17]

RGU-design OE-PCR^†^	Camer and Endoh *et al*. 2018 Novel synthetic DNAs of notifiable dengue, Zika and other flaviviruses: algorithmic OE-PCR	Platform: Linux (Ubuntu) (https://bitbucket.org/dendoh_2/rgu-design-oepcr/src)	Free (distribute on BitBucket)	OE-PCR oligomers can be designed on the limitation of user-determined oligomer length.	Program language Ruby must be installed on the user's computer. Skills for executing Ruby program is needed.	^†^

^†^RGU-design OE-PCR refers to current report of Camer and Endoh *et al*., 2018 of Rakuno Gakuen University, Department of Radiation Biology, School of Veterinary Medicine, Japan.

OE-PCR: Overlap extension-polymerase chain reaction.

It must be noted that the price of oligomers varies greatly depending on the length in base and according to the viral species involved. Owing to this, an algorithm was designed to achieve the maximum length within the lowest cost range in base size. Unfortunately, this is difficult to realize using the currently available reported design algorithms. In the stated design algorithms, the difficulty lies in how to control the length of the oligomer. This is because the setting of overlapping region of the oligomer has been designed first. With the current study, the basic length has been decided to begin with; coupled with a lowered priority on the thermodynamic stability of the overlapping region. This has resulted in fewer problems in the conduct of OE-PCR.

## Conclusion

The ongoing dilemma of largely problematic and imperfectly developed flaviviral immunogens prompted us to responsibly contribute on the need to develop an efficient algorithmic OE-PCR program design for synthetic nucleotide production of select notifiable dengue (1–4), JEV, YF and Zika flaviviruses. To this end, we developed an OE-PCR program that can be utilized as reference tool in the furtherance of flaviviral research. It can be deduced that the program formulation was found to be demonstrably reproducible, cost-effective and efficient in yielding substantial nucleotides of select notifiable flaviviral DNAs, which can be sourced out at the databases of DDBJ/NCBI with the respective accession numbers of LC227563, LC227561, LC227562, and LC227568 (dengue 1–4); LC227560 (JEV); LC227567 (YF); and LC227589 (Zika). The OE-PCR is a reliable method that can be utilized when designed and accurately predicted using algorithmic manipulation. Henceforth, we finally conclude that the DNA primer design using algorithmic OE-PCR is a valuable tool in producing synthetic nucleotide products of pathogenic species of flaviviruses.

## Future perspective

The novel nucleotide sequences generated from this study can be utilized for studies looking to further understand the genomic diversity of a variety of flaviviral strains, including dengue (1–4), YF, JEV and Zika. In addition, clinical trials involving initial utilization of whole nucleotide sequence followed by booster immuno-stimulation of antigenic protein may serve as a starting point for generating long-term immune responses for flaviviral infections such as dengue and Zika, where trials for DNA-based vaccines are ongoing, yet faced with challenging dose-dependency concerns. Further experiments should be conducted to determine whether administration of synthetic nucleotides *in vivo* would result in pathological or immunological response.

**Box 1.**  **The oligomers of the flaviviruses (dengue virus 1–4, Japanese encephalitis, yellow fever and Zika).**

flavi |2|1: ATGTATGCCGATGACACCGCAGGATGGGATACAAGAATCACACTAGAAGACCTAAAAAAT: 60

flavi |2|2: TTGTGTTCTCCTTCCATGTGGTTTGTTACCATTTCTTCATTTTTTAGGTCTTCTAGTGT: 59

flavi |2|3: CATGGAAGGAGAACACAAGAAACTAGCCGAGGCCATTTTCAAACTAACGTACCAAAACA: 59

flavi |2|4: TGTGCCTCTTGGTGTTGGTCTTTGCACACGCACCACCTTGTTTTGGTACGTTAGTTTG: 58

flavi |2|5: CAACACCAAGAGGCACAGTAATGGACATCATATCGAGAAGAGACCAAAGAGGTAGTGGA: 59

flavi |2|6: TGGTGAAAGTATTGAGTCCATAGGTGCCAACTTGTCCACTACCTCTTTGGTCTC: 54

flavi |3|1: ATGTATGCTGATGACACAGCAGGCTGGGACACAAGAATCACTGAGGATGACCTTCAAA: 58

flavi |3|2: TGGTGGGGAGCCATCTGTTCCGTGATCAGTTCCTCATTTTGAAGGTCATCCTCAGT: 56

flavi |3|3: TGGCTCCCCACCACAAGATCCTAGCCAAAGCCATTTTCAAACTAACCTATCAAAACAA: 58

flavi |3|4: TCCCCGCGGTGTGGGTCTGAGGACTTTCACCACTTTGTTTTGATAGGTTAGTTTG: 55

flavi |3|5: CACCGCGGGGAGCGGTGATGGATATCATATCCAGGAAAGACCAAAGAGGTAGTGGA: 56

flavi |3|6: TGGTGAATGTGTTCAAACCATATGTTCCAACTTGTCCACTACCTCTTTGGTCTT: 54

flavi |5|1: ATGTATGCAGATGACACAGCCGGATGGGACACAAGAATAACAGAGGATGATCTTCAGAA: 59

flavi |5|2: CATGTTCAGGTTCCATGATGTCAGTGATTTTGGCCTCATTCTGAAGATCATCCTCTGT: 58

flavi |5|3: CATGGAACCTGAACATGCCCTATTGGCCACGTCAATCTTTAAGCTAACCTACCAAAACA: 59

flavi |5|4: GTTCCATTTTTCGCTGGTCTCTGCACCCTTACTACCTTGTTTTGGTAGGTTAGCTTA: 57

flavi |5|5: AGCGAAAAATGGAACCGTGATGGATGTCATATCCAGACGTGACCAGAGAGGAAGTGG: 57

flavi |5|6: TGGTGAAGGTGTTTAAGCCATAGGTTCCAACCTGTCCACTTCCTCTCTGGTCA: 53

flavi |8|1: ATGTATGCTGATGACACAGCTGGTTGGGACACAAGAATAACAGAAGATGACCTGCACA: 58

flavi |8|2: TGTGTTCAGGGTCCATTTGCTGTATGATCTTTTCCTCATTGTGCAGGTCATCTTCTG: 57

flavi |8|3: AATGGACCCTGAACACAGGCAGTTAGCGAACGCTATATTCAAGCTCACATACCAAAACA: 59

flavi |8|4: TGCCCGTTGGAGTCGGTCGTTGAACTTTGACCACTTTGTTTTGGTATGTGAGCTTG: 56

flavi |8|5: ACTCCAACGGGCACGGTAATGGATATTATATCTAGGAAAGACCAAAGGGGCAGT: 54

flavi |8|6: TGGTGAATGTATTCAGGCCATAAGTTCCCAGTTGTCCACTGCCCCTTTGGTC: 52

flavi |15|1: ATGTACGCAGATGACACTGCTGGCTGGGACACCCGCATTAGTAAGTTTGATCTGGAGAA: 59

flavi |15|2: GTGCCCTTCCTCCATTTGGTTGGTAATCAGAGCTTCATTCTCCAGATCAAACTTACT: 57

flavi |15|3: TGGAGGAAGGGCACAGAACTCTGGCGTTGGCCGTGATTAAATACACATACCAAAACAA: 58

flavi |15|4: TTTTTCCTCCTTCAGCTGGTCTGAGAACCTTCACCACTTTGTTTTGGTATGTGTATTTA: 59

flavi |15|5: CAGCTGAAGGAGGAAAAACAGTTATGGACATCATTTCAAGACAAGACCAGAGAGGGAG: 58

flavi |15|6: TGGTGAATGTGTTGAGAGCATAAGTGACAACTTGTCCACTCCCTCTCTGGTCTTGTC: 57

flavi |25|1: TTCTACGCGGATGACACCGCTGGATGGGACACGCGCATCACAGAGGCAGACCTTGAT: 57

flavi |25|2: TGTGATGTGGGCTCATGTAGTTCAAGATCTCCTGTTCATCATCAAGGTCTGCCTCTG: 57

flavi |25|3: ATGAGCCCACATCACAAAAAACTGGCACAAGCAGTGATGGAAATGACATACAAGAACA: 58

flavi |25|4: TTCCCTCCTGGGGCTGGTCTCAACACTTTCACCACTTTGTTCTTGTATGTCATTTCC: 57

flavi |25|5: GCCCCAGGAGGGAAAGCCTACATGGATGTCATAAGTCGACGAGACCAGAGAGGAT: 55

flavi |25|6: TGGTGATGGTGTTCAGAGCATAAGTCACTACCTGCCCGGATCCTCTCTGGTCTCGT: 56

flavi |27|1: ATGTACGCTGATGACACCGCCGGATGGGACACTAGAATTACCAGAACTGATTTAGAAAA: 59

flavi |27|2: GGTGTTCACCGTCTAGGAGCTCCAGCACCTTGGCTTCATTTTCTAAATCAGTTCTGGT: 58

flavi |27|3: CCTAGACGGTGAACACCGCATGCTCGCTCGAGCCATAATTGAACTGACTTACAGGCACA: 59

flavi |27|4: CTTTCCTTCTGCTGCAGGTCTCATGACCTTGACCACTTTGTGCCTGTAAGTCAGTTCA: 58

flavi |27|5: TGCAGCAGAAGGAAAGACCGTAATGGACGTGATATCAAGAGAAGATCAAAGGGGGAG: 57

flavi |27|6: TCGTGAAAGTGTTAAGGGCATAAGTGACCACCTGTCCACTCCCCCTTTGATCTTCT: 56

**Summary points**

- This project aimed to develop synthetic nucleotides using overlap extension-polymerase chain reaction (OE-PCR), particularly of flaviviral organisms that are notorious for their ability to cause both animal and human diseases.
- A method is provided for generating template designs that could help to address epidemics caused by select flaviviral dengue, Zika and related species.
- Expansion of OE-PCR as a tool in developing templates for viral studies is presented, addressing concern regarding the lack of reported commercial production of artificial nucleotides of these flaviviruses.
- This research has delivered a reproducibly effective and efficient algorithmic OE-PCR design that is suitable for yielding substantial nucleotides of these flaviviruses.
- These synthetic nucleotides could pave the way for future collaborative research exploring the possibility of generating synthetic flaviviral nucleotide-based immunogens.
